# Insight into the Impact of Oxidative Stress on the Barrier Properties of Lipid Bilayer Models

**DOI:** 10.3390/ijms23115932

**Published:** 2022-05-25

**Authors:** Zahra Nasri, Mohsen Ahmadi, Johanna Striesow, Mehdi Ravandeh, Thomas von Woedtke, Kristian Wende

**Affiliations:** 1 Center for Innovation Competence Plasmatis, Leibniz Institute for Plasma Science and Technology, 17489 Greifswald, Germany; mohsen.ahmadi@inp-greifswald.de (M.A.); johanna.striesow@inp-greifswald.de (J.S.); mehdi.ravandeh@inp-greifswald.de (M.R.); woedtke@inp-greifswald.de (T.v.W.); 2 Institute for Hygiene and Environmental Medicine, University Medicine Greifswald, 17475 Greifswald, Germany

**Keywords:** reactive oxygen and nitrogen species, cold physical plasma, cell membrane, supported lipid bilayers, electrochemistry, Langmuir-Blodgett and Schaefer techniques, mass spectrometry

## Abstract

As a new field of oxidative stress-based therapy, cold physical plasma is a promising tool for several biomedical applications due to its potential to create a broad diversity of reactive oxygen and nitrogen species (RONS). Although proposed, the impact of plasma-derived RONS on the cell membrane lipids and properties is not fully understood. For this purpose, the changes in the lipid bilayer functionality under oxidative stress generated by an argon plasma jet (kINPen) were investigated by electrochemical techniques. In addition, liquid chromatography-tandem mass spectrometry was employed to analyze the plasma-induced modifications on the model lipids. Various asymmetric bilayers mimicking the structure and properties of the erythrocyte cell membrane were transferred onto a gold electrode surface by Langmuir-Blodgett/Langmuir-Schaefer deposition techniques. A strong impact of cholesterol on membrane permeabilization by plasma-derived species was revealed. Moreover, the maintenance of the barrier properties is influenced by the chemical composition of the head group. Mainly the head group size and its hydrogen bonding capacities are relevant, and phosphatidylcholines are significantly more susceptible than phosphatidylserines and other lipid classes, underlining the high relevance of this lipid class in membrane dynamics and cell physiology.

## 1. Introduction

Oxidative stress-based therapies, e.g., radiotherapy or photodynamic therapy, are designed to target the metabolic-redox circuits by increasing the intracellular reactive oxygen and nitrogen species (RONS) content [1]. The induced oxidative stress results in notable damage to cellular structures like proteins, nucleic acids, or lipids, forcing the cell to react [2]. A variety of downstream effects is sparked, e.g., cell death via apoptosis, necrosis, or ferroptosis [3], or a fostered immune response via chemotactic signaling and subsequent immune cell differentiation [3,4]. The range of applications for such therapy strategies grows with understanding the underlying mechanisms, including wound management and other skin-related diseases, autoimmune disorders, and cancer [5,6,7]. Cold physical plasma (CPP), a new field of applied redox biology, is an emerging technology in that sector [8]. CPP is a partially ionized gas generated mainly by applying an electric field to a (noble) gas, igniting a discharge consisting of free electrons, RONS, ions, light radiation, and other neutral molecules in a matrix of gas atoms/molecules in the ground state [4,9]. It is assumed that the unique properties of CPP are governed by the spatial distribution and diversity of generated RONS species [10]. The CPP-induced RONS resemble those of other oxidative stress-based therapies in type and density (e.g., ^•^OH, ^1^O_2_, H_2_O_2_, ^•^O_2_^−^, ^•^O, O_3_, ^•^NO, ^•^NO_2_, ONOO^−^). The instruments are small, easy to use, and deposit RONS in a spatially and timely controlled way [11]. Thus, CPP is a proven tool for several biomedical applications [12,13,14,15,16,17,18,19], while current research focuses on cancer treatment [20,21,22,23].

The CPP-induced RONS are produced in the gas phase and, additionally, across the plasma-liquid interface. While the long-lived species diffuse into the tissue layer to interact with cells [24], the short-lived species may react at or close to their point of generation with biomolecules [25,26,27]. The cell membrane as one of the cell’s most vital and functional components representing a dynamic barrier and providing transport and regulatory tasks poses a relevant target for the CPP-produced RONS. Aquaporins and other channels allow the penetration of species like hydrogen peroxide [28,29], while more reactive species are lost assumingly to the reaction with the membrane components, especially the lipids. Accompanying, changes in the barrier properties due to modified molecule polarity and spatial requirements are inevitable [30]. A number of reports addressed this point, hypothesizing a transient poration of the cell membrane, but evidence remains weak [31,32].

Studying the cell membrane’s physical and chemical properties in situ is challenging due to its dynamics, anisotropy, and inhomogeneity. Membranes obtained from exosomes are an alternative approach for investigating biomimetic membranes. However, as they contain the complete cell membrane components, the purification and characterization of the lipids would be a significant challenge [33], plus controlling the composition is impossible. Nanodiscs that have evolved for membrane protein studies in recent years focus more on the protein part than the lipids. Therefore, we preferred supported lipid bilayers (SLBs) for their limited and controllable complexity while still mimicking the structure and essential features of biological membranes [34]. SLBs allow focusing only on the main targets in our study, which are lipids, and make designing bilayers with different lipid compositions and fractions possible [35]. Being reasonably reliable, they have been established to study the structural and chemical modifications of lipids due to the plasma treatment and explore the transport of RONS across the lipid membranes [36,37,38,39,40]. Despite the ubiquity of bilayer asymmetry in biomembranes, existing research is limited to symmetric vesicles. Bilayer asymmetry, or the non-random distribution of different lipids in the bilayer, is a common feature of all membranes, which provides various properties with the two sides of the membrane [41]. The cell bilayer with a thickness of about 4 nm comprises hundreds of different lipids differing in their fatty acid side chains and hydrophilic headgroups. Phosphatidylcholine, sphingomyelin, phosphatidylethanolamine, phosphatidylserine, and cholesterol are the essential lipids of various living cells. As the distribution of the lipids within the biological membrane is highly asymmetric, it is essential to assemble bilayers with a different composition in the base and the top leaflets, which can be achieved by Langmuir−Blodgett (LB)/Langmuir−Schaefer (LS) deposition techniques. In the present study, LB deposition was applied to transfer the first lipid monolayer onto a gold surface, followed by LS deposition for the second one to mimic the asymmetric SLB models [42,43]. This approach allowed us to construct models that mimic the structure and the properties of the cell membrane more closely. For asymmetric bilayer assembly, lipids were selected based on their distribution in the erythrocyte cell membrane. The majority of two choline-containing phospholipids, phosphatidylcholine and sphingomyelin, were localized in the outer leaflet. Two aminophospholipid were predominantly phosphatidylethanolamine, or exclusively phosphatidylserine concentrated in the cytoplasmic half of the bilayer, whereas cholesterol was evenly distributed. Within the inner and outer leaflet, levels of unsaturation varied: the cytoplasmic lipid layer was rich in unsaturated phospholipids compared to the exoplasmic side. This highly asymmetric plasma membrane distribution is typical for most mammalian cells [44]. The obtained asymmetric bilayers were challenged by plasma-produced RONS and tested for changes in permeability. In addition, the role of the headgroup composition on the barrier properties was investigated. Finally, high-resolution liquid chromatography-tandem mass spectrometry (LC-MS^2^) was applied to elucidate the lipid oxidation by CPP [45].

## 2. Results and Discussion

### 2.1. Effect of Plasma-Derived RONS on the Permeability of the Asymmetric Model Lipid Bilayer

**Figure 1:**
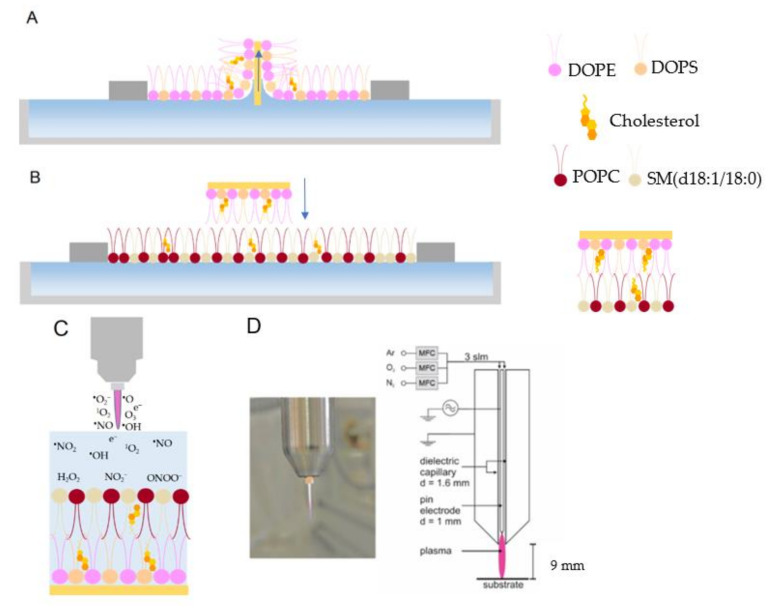
(**A**) Langmuir-Blodgett deposition of the first monolayer (inner leaflet) and (**B**) Langmuir-Schaefer deposition of the second monolayer (outer leaflet) on the surface of the gold to prepare an asymmetric supported lipid bilayer. (**C**) Schematic of the treatment of the supported lipid bilayer by cold physical plasma. (**D**) Image and schematic of the plasma jet kINPen used in this study. DOPE: 1,2-dioleoyl-sn-glycero-3-phosphoethanolamine, DOPS: 1,2-dioleoyl-sn-glycero-3-phospho-L-serine, POPC: 1-palmitoyl-2-oleoyl-sn-glycero-3-phosphocholine, SM (d18:1/18:0): N-stearoyl-D-erythro-sphingosylphosphorylcholine.

1-palmitoyl-2-oleoyl-sn-glycero-3-phosphocholine (POPC), N-stearoyl-D-erythro-sphingosylphosphorylcholine (SM d18:1/18:0), 1,2-dioleoyl-sn-glycero-3-phosphoethanolamine (DOPE), 1,2-dioleoyl-sn-glycero-3-phospho-L-serine (DOPS) and cholesterol were utilized for the preparation of the asymmetric lipid bilayer. The inner leaflet consisting of DOPE: DOPS: cholesterol (55%:35%:10%) was transferred to the surface of the gold electrode using the Langmuir-Blodgett technique (Figure 1A).

**Figure 2:**
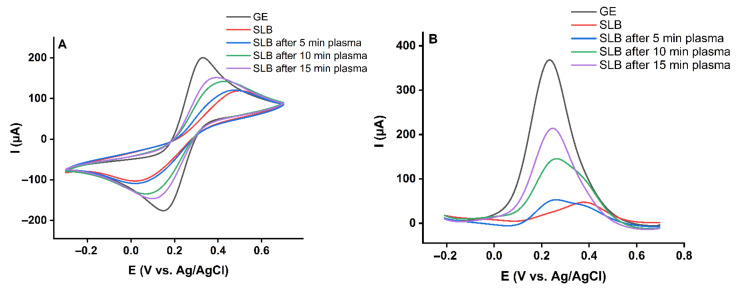
(**A**) Cyclic voltammograms and (**B**) differential pulse voltammograms of 10 mM K_4_[Fe(CN)_6_] in PBS for the gold electrode (GE) and asymmetric gold supported lipid bilayer (SLB) including 10% cholesterol before and after 5, 10, and 15 min plasma treatment.

The outer leaflet, including POPC: SM (d18:1/18:0): cholesterol (45%:45%:10%), was transferred by the Langmuir-Schaefer deposition technique to complete the bilayer (Figure 1B). Both monolayers were deposited at the gel phase (Figure S1). The transfer pressure of the monolayer is one of the most critical parameters dictating the quality of the resulting SLBs [43]. Optimum deposition pressure (Table S1) was determined where the slope of the plot of surface pressure as the function of area per lipid molecule isotherm was at its steepest, and a minor loss in the area with time was observed prior to deposition. This procedure allowed the formation of uniform and stable monolayers with very few defects [46,47]. As a result, membrane-spanning holes are minimized, and lateral cohesion between lipids is enhanced, facilitating the analysis of CPP-lipid interaction. Since cholesterol leads to a more fluid gel phase during deposition, defect frequency in the SLB increases [48]. Based on that, 10% cholesterol in the SLB was the maximum concentration that could be achieved while still forming a high-quality bilayer suitable for electrochemical analysis experiments. Electrochemical measurements confirmed the successful transfer of the lipid bilayer (Figure 2). The cyclic voltammogram (CV) and differential pulse voltammogram (DPV) of the redox probe (10 mM K_4_[Fe(CN)_6_]) in the presence of gold supported lipid bilayer was nearly flat and showed no peak current. This indicated the complete transfer of the lipids and a successful formation of the bilayer on the gold electrode. Blocking the access of the redox probe to the electrode surface prevented the electron transfer reaction and, subsequently, led to the diminished current. CPP treatment for 5 min did not increase the current significantly, which showed the asymmetric model lipid bilayer was stable against produced RONS in short treatment times. However, the probe’s electron transfer through the treated lipids was more effortless than the untreated one, which led to a slight decrease in the potential. Increasing the treatment time to 10 min and 15 min significantly raised the lipid bilayer permeability (Figure 2) due to the prolonged attack of reactive species. Given the fact that the liquid bulk created a significant distance between the gas-liquid interface and the liquid-SLB interface, short-lived primary species generated in the plasma effluent can be excluded. Alongside previous reports [49,50], it is assumed that secondary/tertiary reactive species such as ^•^OH or ONOO^–^ are responsible.

### 2.2. Effect of Cholesterol on the Barrier Properties of the Model Lipid Bilayer

**Figure 3:**
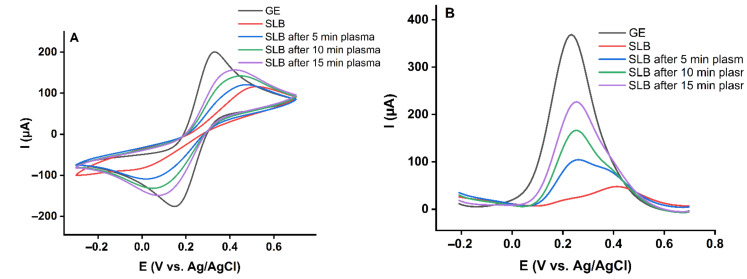
(**A**) Cyclic voltammograms and (**B**) differential pulse voltammograms of 10 mM K_4_[Fe(CN)_6_] in PBS for the gold electrode (GE) and asymmetric gold supported lipid bilayer (SLB) with no cholesterol before and after 5, 10, and 15 min plasma treatment.

It is discussed that CPP treatment is selective towards cancer cells to some extent since these often feature higher base levels of RONS and can be more easily driven into a pro-oxidant condition. In addition, healthy cells are more efficient in neutralizing RONS [51]. A further difference concerns the presence of cholesterol, a molecule essential for maintaining the balance between fluidity and rigidity in the cell membrane. In healthy cell membranes, the cholesterol levels are higher than in cancer cell counterparts, suggesting a role in RONS handling [52]. For this purpose, cholesterol was excluded from the model lipid bilayer. The inner leaflet made of DOPE: DOPS (60%:40%) and the outer leaflet included POPC: SM (d18:1/18:0) (50%:50%) were prepared and treated with plasma for 5, 10, and 15 min. The recorded cyclic voltammograms and differential pulse voltammograms after plasma treatment are shown in Figure 3. The data underline the importance of cholesterol in protecting the lipid bilayer by attenuating lipid oxidation and subsequently defect or pore formation. However, 10% cholesterol rescues the bilayer from oxidative damage only for shorter treatment times (5 min). This result is in line with simulations on the protective role of cholesterol [53], and predictions suggest that by raising the cholesterol amount, the barrier properties of the SLBs against RONS will elevate [54]. It can be assumed that structurally tense molecule cholesterol is a good target for ROS, especially singlet oxygen ^1^O_2_ that can form endoperoxide intermediates. The oxidation products do not possess a protective role. After cholesterol has been consumed, membrane permeability increased independently of the presence of cholesterol (Figure 2 and Figure 3).

### 2.3. Effect of Lipid Structure on the Barrier Properties of the Model Lipid Bilayers

**Figure 4:**
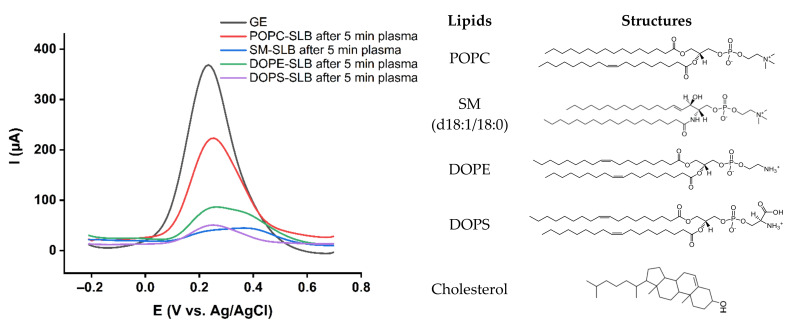
Differential pulse voltammograms of 10 mM K_4_[Fe(CN)_6_] in PBS for the gold electrode (GE) and gold supported lipid bilayers from POPC (phosphatidylcholine class), SM (d18:1/18:0) (sphingosylphosphorylcholine class), DOPE (phosphoethanolamine class), and DOPS (phosphoserine class).

The membrane composition essentially controls the properties of the lipid bilayer. Lipids exhibit different headgroup structures, which vary in size, charge, polarity, and hydrogen bonding capacities, strongly impacting the lipid bilayer characteristics [55]. To fully comprehend the membrane’s properties, the intermolecular interactions determining the behavior of the lipid bilayer should be highlighted. For this purpose, the major constituent lipids of the membrane were studied. In this regard, symmetric lipid bilayers from POPC (phosphatidylcholine class), SM (d18:1/18:0) (sphingomyelin class), DOPE (phosphatidylethanolamine class), and DOPS (phosphatidylserine class) were transferred onto the gold surface and subjected to plasma treatment (Figure 4). The highest increase in permeability was observed for POPC lipid bilayers while SM (d18:1/18:0) rich bilayers remain undisturbed. Both POPC and SM (d18:1/18:0) belong to the exoplasmic membrane leaflet, and both bear a choline headgroup yet behave entirely differently. Besides the higher saturation level in SM (d18:1/18:0), sphingomyelin lipid class forms extensive intramolecular and intermolecular hydrogen bondings in the membrane environment via their amide and hydroxyl groups that stabilize the bilayer. This hydrogen bonding network in the sphingomyelin bilayer behaves as a barrier against reactive species and prevents or reduces permeability changes due to the plasma treatment [49,56,57].

DOPE lipid bilayer also showed improved barrier properties against reactive species compared to POPC. Phosphatidylethanolamine and phosphatidylcholine are the most common phospholipids in eukaryotes. Both are glycerophospholipids, but phosphatidylethanolamine has an amine headgroup, which is smaller than the choline headgroup of phosphatidylcholine. The smaller headgroup of phosphatidylethanolamine allows closer packing of the lipid hydrocarbon chains. The tighter packing increases barrier properties and decreases film permeability [58]. While the amine headgroup of phosphatidylethanolamine molecules can form hydrogen bonding directly to each other, phosphatidylcholine molecules interact indirectly via hydrogen bonding with water molecules [55,59]. These factors lead to a more closely packed phosphatidylethanolamine membrane containing less water and restricted RONS access, enhancing the barrier properties. The results indicate that DOPS lipid bilayers behave as effective barriers against reactive species (Figure 4), showing a similar resistance as SM (d18:1/18:0) bilayers. It cannot be fully excluded that the negatively charged serine head group repels the ferrocyanide redox probe (charge: −4) during the permeability measurements, leading to overestimating the barrier properties. However, to maintain the repulsing effect, the lipid must be intact.

As POPC was identified as the most susceptible lipid to plasma-produced reactive species, more experiments were done to find how the addition of sphingomyelin and cholesterol changes the barrier properties of the exoplasmic side of the cell membrane. To this aim, symmetric bilayers with lipid composition of POPC: SM (d18:1/18:0) (50%:50%) and POPC: SM (d18:1/18:0): cholesterol (45%:45%:10%) were prepared, and tested for resilience against plasma treatment.

**Figure 5:**
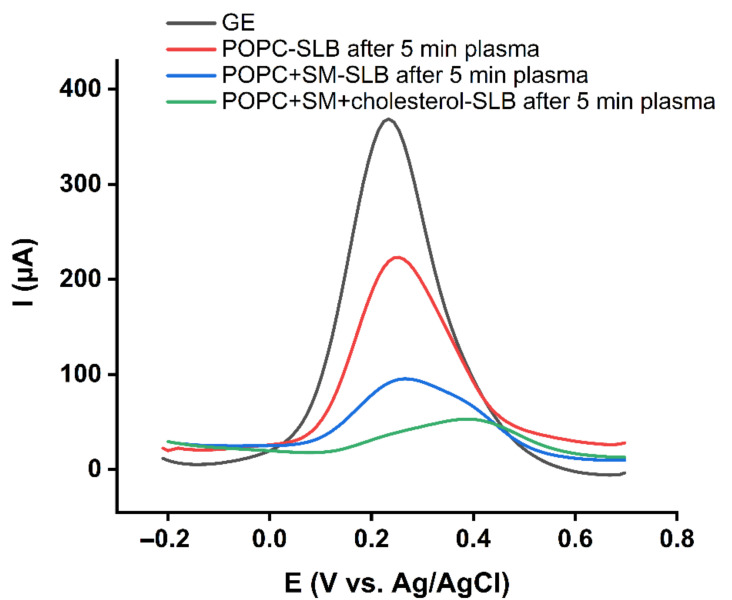
Differential pulse voltammograms of 10 mM K_4_[Fe(CN)_6_] in PBS recorded for the gold electrode (GE), and symmetric lipid bilayers from POPC, POPC + SM, and POPC + SM + 10% cholesterol after 5 min plasma treatment.

As shown in Figure 5, adding SM (d18:1/18:0) and cholesterol to the POPC lipid bilayers increased stability against reactive species via the formation of additional hydrogen bonds. The association between sphingomyelin and cholesterol significantly enhances the direct intermolecular hydrogen bonding and, consequently, the barrier properties of the lipid bilayers [56]. Similar compositions and such interactions can also be found in the cell membranes lipid rafts, which are more ordered and tightly packed than the surrounding bilayer [60].

### 2.4. Effect of Long-Lived Species on the Model Lipid Bilayer

**Figure 6:**
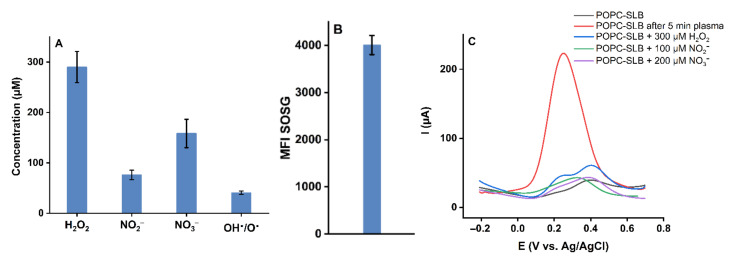
(**A**) Concentration of reactive species produced after 5 min plasma treatment of PBS (pH 7.4), (**B**) ^1^O_2_ was detected using the SOSG assay, (**C**) Differential pulse voltammograms of 10 mM K_4_[Fe(CN)_6_] in PBS recorded for the POPC lipid bilayer after 5 min plasma treatment, and after 5 min incubation of the bilayer with selected long-lived RONS, the added concentrations matched with results from 5 min plasma treatment.

CPP produces different types and amounts of short- and long-lived reactive species, diffusing in the bulk liquid to reach the lipid bilayer and interact with it. The generation of selected reactive species was determined to curtail which plasma-produced reactive species are responsible for the observed changes in the lipid bilayer permeability (Figure 6A). As expected, H_2_O_2_, NO_2_^−^, and NO_3_^−^ are the major long-lived species generated in phosphate-buffered saline. Moreover, a moderate formation of atomic/OH^•^ was observed, and the singlet oxygen sensor green confirmed the production of ^1^O_2_ (Figure 6B). Control experiments incubating the POPC lipid bilayer with concentration matched amounts of deposited long-lived species confirmed that H_2_O_2_, NO_2_^−^, and NO_3_^−^ do not spark changes in model bilayer permeability (Figure 6C). Consequently, short-lived reactive species appear responsible. While atomic oxygen or hydroxyl radicals cannot penetrate the liquid bulk due to their reactivity, it might be speculated that a secondary formation takes place, as was suggested by Verlackt et al. [61]. Similarly, ozone (from dissolved molecular oxygen and atomic oxygen) or peroxynitrite (from H_2_O_2_ and NO_2_^−^ at the gas-liquid interface) might be relevant for the observed destruction of the lipid film. Since specific sensors were not available, lipid oxidation was analyzed by high-resolution LC-MS^2^.

### 2.5. Plasma-Induced Chemical Modifications of the Model Lipids

**Figure 7:**
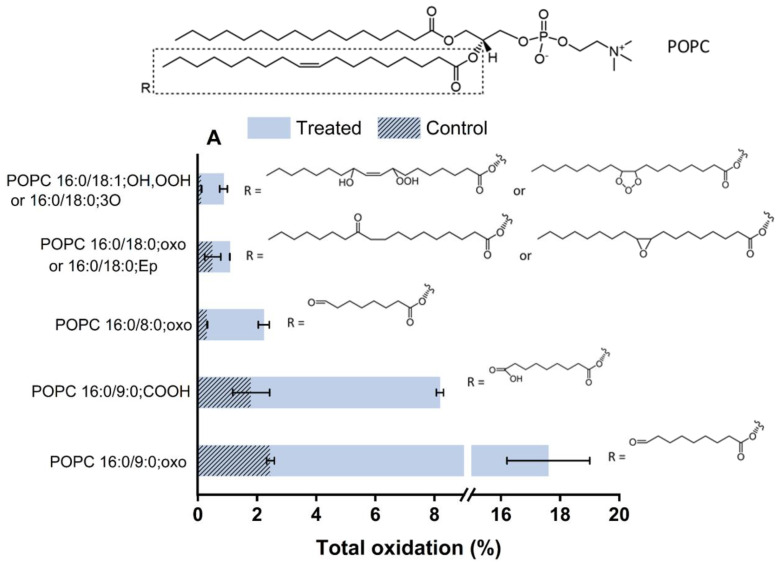

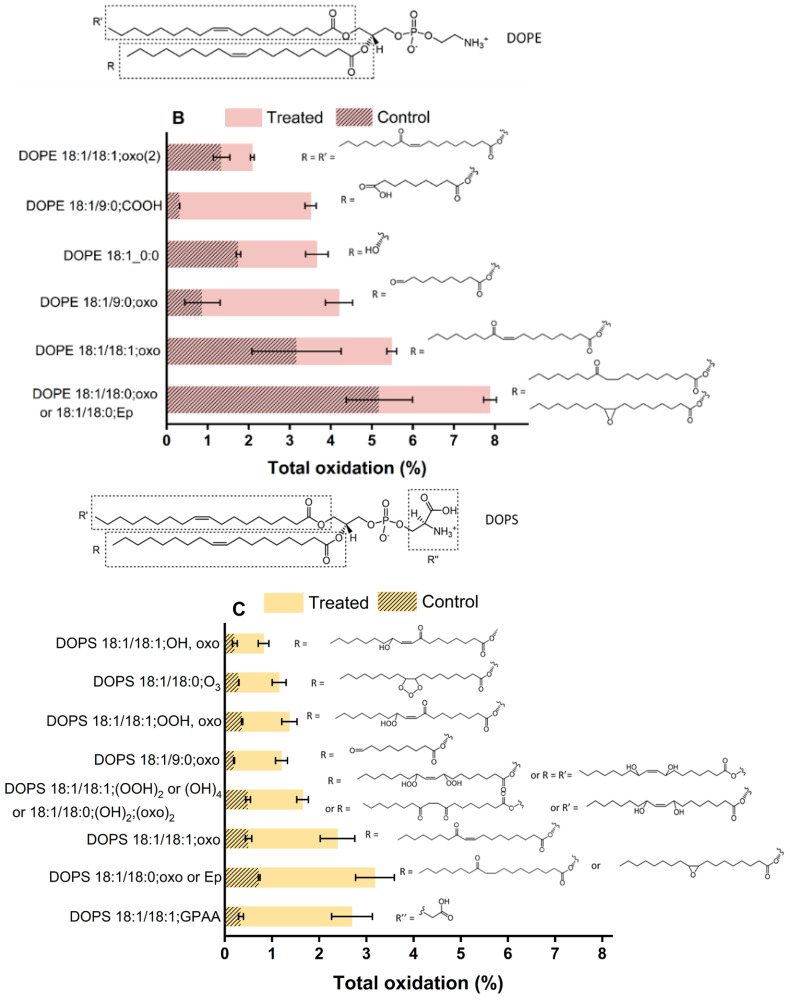

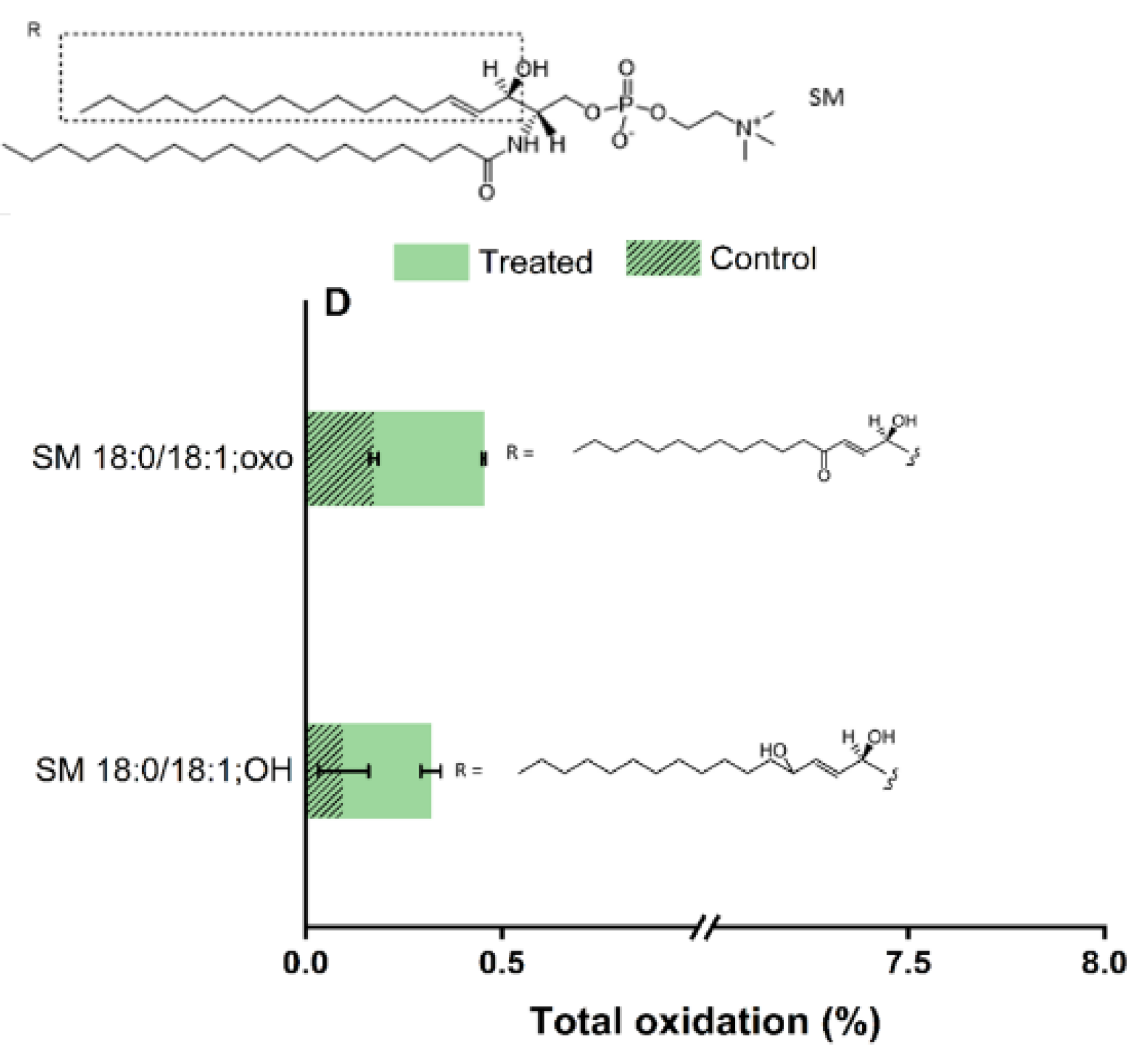
Oxidation products of (**A**) POPC, (**B**) DOPE, (**C**) DOPS, and (**D**) SM (d18:1/18:0) after 15 min plasma treatment compared to the auto-oxidation (control). The total oxidation is calculated as the peak area of each lipid oxidation product divided by the peak area of parent lipid in the same sample. EP: epoxy, GPAA: glycerophospholipid acetic acid.

After excluding the long-lived species from relevance, the impact of short-lived RONS on the model lipids was investigated by LC-MS^2^ experiments. Challenging the lipids for 15 min by plasma treatment revealed a wide range of lipid oxidation products with different oxidation states and positions. Figure 7 presents the type and total percentage of oxidations induced by plasma on the components of the asymmetric lipid bilayer. Not all constituents of the bilayer were oxidized to the same extent, and the type and amount of observed oxidations changed with the chemical composition of the lipids.

The exoplasmic side of the bilayer, in the model POPC and SM (d18:1/18:0), pose initial targets for the attacking species. While POPC showed a range of oxidation products, SM (d18:1/18:0) was not modified considerably. Besides the structural difference of the Z-configured double bond of SM with an α-hydroxyl group reducing electron density and subsequently reactivity of the allylic position, the ability to form extensive hydrogen bondings inhibited reactive species penetration to the tail of the lipids and led to negligible oxidation. In contrast, when looking at the cytoplasmic leaflet, DOPE and DOPS showed numerous oxidative modifications. The corresponding extracted ion chromatograms for the products and the MS/MS fragment spectra are presented in Figure S2. Due to the presence of isobaric molecules and missing indicative fragmentation products, some structures in Figure 7 remain ambiguous and are marked with “or”. The observed lipid oxidation products reflect mainly an attack on the unsaturated bonds and the allylic H-atom of the lipid tails, followed by an abstraction of hydrogen atoms and the addition of reactive species with an O and O-O motif, such as molecular oxygen, atomic oxygen, ozone, and OH radicals, yielding to hydroperoxides, hydroxyl groups, oxo groups, or oxygen-containing rings. If the oxidative reaction proceeds further, chain breaks occur by subsequent rearrangements (Hock cleavage, homolytic β-scission, etc.), leading to the formation of aldehyde and carboxylic acid products [50] accompanied by the release of short alcohols or aldehydes (e.g., 4-hydroxy nonenal/4-HNE, malondialdehyde/MDA). The residual truncated lipids, such as 1-palmitoyl-2-(9’-oxo-nonanoyl)-sn-glycero-3-phospho derivatives (Poxno-Px) or 1-palmitoyl-2-azelaoyl-sn-glycero-3-phospho derivatives (PAze-Px, x = C, E, or S), have shorter, more polar lipid tails, that yield to losses in the barrier properties. Even lipid tails that carry only one additional oxygen (e.g., DOPE 18:1/18:1, oxo) reduce the crystallinity of the membrane. Of note, considerable head group oxidations were not observed in the current conditions, indicative of the absence or a low concentration of hypochlorite OCl^−^, which can evolve from atomic oxygen and chloride ions.

The obtained LC-MS^2^ results demonstrated that the plasma-induced oxidation led to attaching one or more oxygen atoms to the unsaturated lipid chains yielding more hydrophilic oxidized products that are in agreement with previous observations [49,50]. The biochemical properties of the products underpin the observed changes in membrane permeability: the oxidized tails with more polar character migrate to the headgroup at the water interface, leading to the increase in the molecular area of the oxidized lipid and the decrease of the bilayer thickness, consequently affecting the fluidity and permeability of the membrane [62,63,64,65]. It has been accepted that the oxidation of lipids to the shortened chain products and the addition of a hydrophilic group to it perturbs the hydrophobic membrane and promotes protrusion formation in the lipid bilayer, which can be considered as a precursor for pore formation. Increasing the concentration of truncated-chain lipids leads to micelle formation. Desorption of such micelles paves the way to the pore formation in the lipid bilayer, membrane disintegration, and the observed increase in permeability [49,66,67]. In vivo, additional signaling events can be triggered by the oxidized lipids or the released fragments (especially 4-HNE), amplifying the physiological response to the plasma treatment [68].

## 3. Materials and Methods

### 3.1. Materials

1-palmitoyl-2-oleoyl-sn-glycero-3-phosphocholine (16:0-18:1 PC (POPC)), N-stearoyl-D-erythro-sphingosylphosphorylcholine (18:0 SM (d18:1/18:0)), 1,2-dioleoyl-sn-glycero-3-phosphoethanolamine (18:1 (Δ9-Cis) PE (DOPE)), 1,2-dioleoyl-sn-glycero-3-phospho-L-serine (sodium salt) (18:1 PS (DOPS)) and cholesterol (ovine wool, 98%) were from Avanti Polar Lipids and purchased from Otto Nordwald (Otto Nordwald GmbH, Hamburg, Germany) and used without further purification. Chloroform and ethanol were HPLC grade and purchased from Carl Roth (Carl Roth GmbH + Co. KG, Karlsruhe, Germany). Methanol (99.95%) was from Th. Geyer (Th. Geyer GmbH & Co. KG, Berlin, Germany). Potassium hexacyanoferrate(II) trihydrate was purchased from Sigma-Aldrich (Sigma-Aldrich Chemie GmbH, Taufkirchen, Germany). The water used for cleaning and measurements was purified with an ultrapure water MilliQ (Milli-Q^®^ Merck KGaA, Darmstadt, Germany). All other reagents were of analytical grade and used without further purification.

### 3.2. Langmuir-Blodgett (LB) and Langmuir-Schaefer (LS) Deposition

Surface pressure-area isotherms (π-A isotherms) were recorded using a Langmuir trough (KSV NIMA, LOT-QuantumDesign GmbH, Darmstadt, Germany) equipped with two hydrophilic barriers and a platinum Wilhelmy plate in a nitrogen atmosphere at 22 °C. Lipid bilayers were prepared on Au(111) (arrandee metal GmbH + Co. KG, Werther, Germany) substrates using a combination of LB and LS depositions. The asymmetric lipid bilayers were produced when the lipids deposited in the LB and LS steps were different. The Langmuir trough was cleaned with ethanol before being filled with ultrapure water. The cleanliness of the water phase was checked before and after the Au substrate was placed in the trough by closing the barrier and monitoring the surface pressure at zero. The Au (111) substrate was flame-annealed, cleaned in the piranha solution, and transferred with a drop of water into the trough [69]. A sufficient quantity of 1 mg mL^−1^ lipid solution of the first monolayer (inner leaflet) was dispersed onto the surface of the water subphase in a dropwise manner. The lipid solvent was allowed to evaporate for 20 min, and then the barriers were closed to obtain the target pressure for the lipid sample. The Au substrate was raised vertically through the monolayer film at a speed of 2 mm min^−1^, while the pressure was maintained at the target pressure (Figure 1A) and then dried in argon for 30 min. A transfer ratio of 1.0 ± 0.1 calculated by the instrument from the area that the barriers move to compensate for the loss of lipids divided by the surface area of the gold substrate moved through the monolayer was accepted as a successful transfer of the lipid to the substrate. After that, the first monolayer in the trough was replaced with the second one (outer leaflet) and was compressed to the appropriate target pressure. A Langmuir-Schaeffer (horizontal dip) was then performed at a speed of 0.5 mm min^−1^. The Au substrate covered by the inner leaflet monolayer was horizontally brought into contact with the outer leaflet monolayer, which was spread and compressed to the target surface pressure on the trough (Figure 1B). The electrode was then slowly lifted upwards to complete the bilayer. The Au substrate was placed immediately into the electrochemical cell (Figure 1C). Target Pressures for the transfer of monolayers with different lipid compositions are presented in Table S1.

### 3.3. Electrochemical Measurements

Electrochemical measurements were carried out by a bipotentiostat AUTOLAB PGSTAT302N (Deutsche METROHM GmbH & Co. KG, Filderstadt, Germany). All experiments were performed using a three-electrode system consisting of a leakless Ag/AgCl miniature reference electrode, a platinum wire auxiliary electrode (eDAQ Europe, Warszawa, Poland) and a gold substrate supported lipid bilayer working electrode.

### 3.4. Plasma Source

The kINPen11 (neoplas tools GmbH, Greifswald, Germany) was used as a well-characterized plasma source running at 1.1 W and a frequency of 1 MHz [70,71] (Figure 1D). Argon was used as feed gas at a flux of 3.0 standard liters per minute (slm). The gold-supported lipid bilayer electrode was placed at the bottom of the electrochemical cell, which was filled with 2.0 mL of phosphate-buffered saline (PBS) pH 7.4 solution. The height of the PBS on the lipid bilayer was 3 cm, and plasma treatment was performed at a distance of 9 mm between the jet nozzle and the buffer surface for 5, 10, and 15 min. The electrochemical cell was connected to a peristaltic pump to top up the evaporated water during plasma treatment and keep the buffer volume constant.

### 3.5. Reactive Species Quantification

The terephthalic acid assay was used to quantify atomic oxygen/hydroxyl radicals [72]. The produced 2-hydroxyl terephthalic was determined at the fluorescence excitation of 360 nm and emission of 465 nm using the spectrophotometer (Infinite M200 Pro plate reader, Tecan Group Ltd., Switzerland). Singlet oxygen was determined by the singlet oxygen sensor green reagent (SOSG, Invitrogen, Waltham, MA, USA). The fluorescence was measured at excitation/emission maxima of 504/525 nm [73]. The amount of produced hydrogen peroxide was analyzed using the Amplex^®^ UltraRed reagent stock (Invitrogen, USA) according to the previously reported protocols [74]. The fluorescence was measured at excitation/emission of 530/590 nm. As previously outlined, nitrite and nitrate were determined by ion chromatography (ICS-5000, Dionex Corp., Sunnyvale, CA, USA) [75]. The analytes were detected by conductivity and UV detection.

### 3.6. Lipid Extraction

After the plasma treatment, the lipid bilayer was washed with PBS gently using a peristaltic pump to remove excess reactive species. After that, a volume of 1 mL methyl tert-butyl ether (MTBE) solution was added to the supported lipid bilayer at the bottom of the electrochemical cell. After shaking the solution for 15 min, aqueous and organic phases appeared. Then, the MTBE phase was separated and removed under a constant nitrogen stream. Finally, the obtained lipid film was kept at −80 ^◦^C.

### 3.7. LC-MS^2^ Analysis

Lipid films were dissolved with a freshly prepared mixture of chloroform:methanol:isopropanol (1:2:4, *v*:*v*:*v*) and separated on a Vanqiush UHPLC system (Thermo Fisher, Germany) equipped with an iHILIC Fusion column (150 × 2.1 mm, 1.8 µM) that was connected to a pre-column (0.2 µm, both obtained from HILICON). The mobile phase consisted of ammonium acetate buffer (35 mM, pH 5.75)/acetonitrile (A, 95/5, *v*/*v*) and acetonitrile (B; 100). The column chamber temperature was 40 °C. Lipids were separated on the following 27 min long gradient: equilibration from 0–0.1 min 98% B, 0.1–0.5 min hold 98% B, 0.5–15 min decrease to 55% B, 15–20 min hold 55% B, increase to 98% B from 20–22 min, hold until 27 min. The applied flow rate was 300 µL/min.

Lipids were detected on a QExactive Plus high-resolution mass spectrometer operated in data-dependent acquisition mode (top 10). The resolution in MS1 was set to 70.000 with a maximum ion time (IT) of 100 ms and an automatic gain control (AGC) target of 1e6. The scan range was from 200–900 m/z. In MS, the resolution was set to 17.500 with a maximum IT of 50 ms and an AGC target of 1e5. Stepped collision energy was applied from 22–23%.

### 3.8. Identification of Lipid Peroxidation Products

Lipid peroxidation products (LPPs) were manually detected in the chromatograms in negative polarity. MS^2^ fragmentation spectra and retention times were checked for correct annotation (Tables S2–S5). LPPs were annotated according to the proposed nomenclature from the LIPID MAPS consortium [76].

## 4. Conclusions

The present study investigated the effect of RONS produced from CPP on the permeability of different asymmetric lipid bilayers using electrochemical detection. The importance of cholesterol in protecting the lipid bilayers against oxidative events and subsequent losses in barrier function could be confirmed. Predominantly the head group characteristics such as charge, size, and hydrogen bonding capacity affected the resilience and barrier properties of the lipid layers during CPP treatment. Phosphatidylcholine based lipid bilayer was most sensitive since the large choline head group impedes tight molecule packing facilitating RONS attack. Phosphatidylethanolamine, Phosphatidylserine, and sphingomyelin-based lipid layers were more robust. Inter and intramolecular hydrogen bonding networks in sphingomyelin and phosphatidylethanolamine led to closely packed lipid films with better barrier properties toward reactive species. These results were confirmed by the significant oxidation of POPC (phosphatidylcholine class) observed in LC-MS^2^ analysis compared to the other lipids, which imposed considerable changes on the biophysical properties of the bilayer, resulting in the increase of film permeability to RONS.

As solely the effect on the major lipids of the membrane was investigated in this study, future efforts will include the analysis of oxidative stress impact on the functionality of membrane proteins as critical components of the cell membrane. A number of recent papers showed oxidative modifications in isolated proteins [26,77,78], suggesting that in the light of the current finding, a competitive situation may arise in a model containing both biomolecules impeding predictions. Tethered lipid bilayer membranes [79] and nanodiscs [80] are two approaches that enable the analysis of membrane proteins in a physiologically relevant environment possible. As in these methods, the embedded proteins in lipid membranes could be immobilized on the surface; they can benefit from the surface-sensitive biophysical characterization techniques to further investigate the impact of oxidative stress on membrane proteins.

## Data Availability

Data will be available on request.

## Supplementary Materials

The following supporting information can be downloaded at: https://www.mdpi.com/article/10.3390/ijms23115932/s1.

## References

[1] Wang K., Jiang J., Lei Y., Zhou S., Wei Y., Huang C. (2019). Targeting Metabolic–Redox Circuits for Cancer Therapy. Trends Biochem. Sci..

[2] Manda G., Isvoranu G., Comanescu M.V., Manea A., Butuner B.D., Korkmaz K.S. (2015). The redox biology network in cancer pathophysiology and therapeutics. Redox Biol..

[3] Zhang H., Wang L., Chu Y. (2019). Reactive oxygen species: The signal regulator of B cell. Free Radic. Biol. Med..

[4] Weltmann K.D., von Woedtke T. (2017). Plasma medicine-current state of research and medical application. Plasma Phys. Control. Fusion.

[5] Gorrini C., Harris I.S., Mak T.W. (2013). Modulation of oxidative stress as an anticancer strategy. Nat. Rev. Drug Discov..

[6] Liu Z., Zhou T., Ziegler A.C., Dimitrion P., Zuo L. (2017). Oxidative Stress in Neurodegenerative Diseases: From Molecular Mechanisms to Clinical Applications. Oxid. Med. Cell. Longev..

[7] Forrester S.J., Kikuchi D.S., Hernandes M.S., Xu Q., Griendling K.K. (2018). Reactive Oxygen Species in Metabolic and Inflammatory Signaling. Circ. Res..

[8] Von Woedtke T., Schmidt A., Bekeschus S., Wende K., Weltmann K.-D. (2019). Plasma Medicine: A Field of Applied Redox Biology. In Vivo.

[9] Khalili M., Daniels L., Lin A., Krebs F.C., E Snook A., Bekeschus S., Bowne W.B., Miller V. (2019). Non-thermal plasma-induced immunogenic cell death in cancer. J. Phys. D. Appl. Phys..

[10] Cheng H., Xu J., Li X., Liu D., Lu X. (2020). On the dose of plasma medicine: Equivalent total oxidation potential (ETOP). Phys. Plasmas.

[11] Wende K., von Woedtke T., Weltmann K.-D., Bekeschus S. (2018). Chemistry and biochemistry of cold physical plasma derived reactive species in liquids. Biol. Chem..

[12] Bekeschus S., Brüggemeier J., Hackbarth C., von Woedtke T., Partecke L.-I., van der Linde J. (2017). Platelets are key in cold physical plasma-facilitated blood coagulation in mice. Clin. Plasma Med..

[13] Kalghatgi S.U., Fridman G., Cooper M., Nagaraj G., Peddinghaus M., Balasubramanian M., Vasilets V., Gutsol A.F., Fridman A., Friedman G. (2007). Mechanism of Blood Coagulation by Nonthermal Atmospheric Pressure Dielectric Barrier Discharge Plasma. IEEE Trans. Plasma Sci..

[14] Bekeschus S., Schmidt A., Weltmann K.-D., von Woedtke T. (2016). The plasma jet kINPen – A powerful tool for wound healing. Clin. Plasma Med..

[15] Dobrynin D., Fridman G., Friedman G., Fridman A. Physical mechanisms of plasma assisted wound healing: Production and delivery of active species, Demanovska dolina, Slovakia, Demanovska dolina, Slovakia, 2011. http://enviro.fmph.uniba.sk/nato/pdfs/O/o16.pdf.

[16] Haertel B., von Woedtke T., Weltmann K.-D., Lindequist U. (2014). Non-Thermal Atmospheric-Pressure Plasma Possible Application in Wound Healing. Biomol. Ther..

[17] Liu D., Zhang Y., Xu M., Chen H., Lu X., Ostrikov K. (2020). Cold atmospheric pressure plasmas in dermatology: Sources, reactive agents, and therapeutic effects. Plasma Process. Polym..

[18] Heinlin J., Isbary G., Stolz W., Morfill G., Landthaler M., Shimizu T., Steffes B., Nosenko T., Zimmermann J., Karrer S. (2010). Plasma applications in medicine with a special focus on dermatology. J. Eur. Acad. Dermatol. Venereol..

[19] Daeschlein G., Scholz S., von Woedtke T., Junger M. (2012). Cold plasma antisepsis for skin and wounds: A new antimicrobial concept in dermatology. Exp. Dermatol..

[20] von Woedtke T., Reuter S., Masur K., Weltmann K.D. (2013). Plasmas for medicine. Phys. Rep..

[21] Keidar M., Yan D., Beilis I.I., Trink B., Sherman J.H. (2018). Plasmas for Treating Cancer: Opportunities for Adaptive and Self-Adaptive Approaches. Trends Biotechnol..

[22] Yan D., Sherman J.H., Keidar M. (2016). Cold atmospheric plasma, a novel promising anti-cancer treatment modality. Oncotarget.

[23] Semmler M.L., Bekeschus S., Schäfer M., Bernhardt T., Fischer T., Witzke K., Seebauer C., Rebl H., Grambow E., Vollmar B. (2020). Molecular Mechanisms of the Efficacy of Cold Atmospheric Pressure Plasma (CAP) in Cancer Treatment. Cancers.

[24] Hirst A.M., Frame F.M., Arya M., Maitland N.J., O’Connell D. (2016). Low temperature plasmas as emerging cancer therapeutics: The state of play and thoughts for the future. Tumor Biol..

[25] Wenske S., Lackmann J.-W., Busch L.M., Bekeschus S., von Woedtke T., Wende K. (2021). Reactive species driven oxidative modifications of peptides—Tracing physical plasma liquid chemistry. J. Appl. Phys..

[26] Wenske S., Lackmann J.-W., Bekeschus S., Weltmann K.-D., Von Woedtke T., Wende K. (2020). Nonenzymatic post-translational modifications in peptides by cold plasma-derived reactive oxygen and nitrogen species. Biointerphases.

[27] Bruno G., Wenske S., Lackmann J.-W., Lalk M., Von Woedtke T., Wende K. (2020). On the Liquid Chemistry of the Reactive Nitrogen Species Peroxynitrite and Nitrogen Dioxide Generated by Physical Plasmas. Biomolecules.

[28] Yan D., Xiao H., Zhu W., Nourmohammadi N., Zhang L.G., Bian K., Keidar M. (2017). The role of aquaporins in the anti-glioblastoma capacity of the cold plasma-stimulated medium. J. Phys. D Appl. Phys..

[29] Bauer G., Sersenová D., Graves D.B., Machala Z. (2019). Cold Atmospheric Plasma and Plasma-Activated Medium Trigger RONS-Based Tumor Cell Apoptosis. Sci. Rep..

[30] Yusupov M., Wende K., Kupsch S., Neyts E.C., Reuter S., Bogaerts A. (2017). Effect of head group and lipid tail oxidation in the cell membrane revealed through integrated simulations and experiments. Sci. Rep..

[31] Vijayarangan V., Delalande A., Dozias S., Pouvesle J.-M., Robert E., Pichon C. (2020). New insights on molecular internalization and drug delivery following plasma jet exposures. Int. J. Pharm..

[32] LeDuc M., Guay D., Leask R., Coulombe S. (2009). Cell permeabilization using a non-thermal plasma. New J. Phys..

[33] Skotland T., Sandvig K., Llorente A. (2017). Lipids in exosomes: Current knowledge and the way forward. Prog. Lipid Res..

[34] Giwa A., Hasan S.W., Yousuf A., Chakraborty S., Johnson D., Hilal N. (2017). Biomimetic membranes: A critical review of recent progress. Desalination.

[35] Luchini A., Vitiello G. (2020). Mimicking the Mammalian Plasma Membrane: An Overview of Lipid Membrane Models for Biophysical Studies. Biomimetics.

[36] Maheux S., Frache G., Thomann J.S., Clément F., Penny C., Belmonte T., Duday D. (2016). Small unilamellar liposomes as a membrane model for cell inactivation by cold atmospheric plasma treatment. J. Phys. D Appl. Phys..

[37] Hong S.-H., Szili E.J., A Jenkins A.T., Short R.D. (2014). Ionized gas (plasma) delivery of reactive oxygen species (ROS) into artificial cells. J. Phys. D Appl. Phys..

[38] Ki S.H., Park J.K., Sung C., Lee C.B., Uhm H., Choi E.H., Baik K.Y. (2016). Artificial vesicles as an animal cell model for the study of biological application of non-thermal plasma. J. Phys. D Appl. Phys..

[39] Tero R., Suda Y., Kato R., Tanoue H., Takikawa H. (2014). Plasma irradiation of artificial cell membrane system at solid–liquid interface. Appl. Phys. Express.

[40] Van der Paal J., Hong S.-H., Yusupov M., Gaur N., Oh J.-S., Short R.D., Szili E.J., Bogaerts A. (2019). How membrane lipids influence plasma delivery of reactive oxygen species into cells and subsequent DNA damage: An experimental and computational study. Phys. Chem. Chem. Phys..

[41] Fadeel B., Xue D. (2009). The ins and outs of phospholipid asymmetry in the plasma membrane: Roles in health and disease. Crit. Rev. Biochem. Mol. Biol..

[42] Bartlett P.N. (2008). Bioelectrochemistry: Fundamentals, Experimental Techniques, and Applications.

[43] Kurniawan J., De Souza J.F.V., Dang A.T., Liu G.-Y., Kuhl T.L. (2018). Preparation and Characterization of Solid-Supported Lipid Bilayers Formed by Langmuir–Blodgett Deposition: A Tutorial. Langmuir.

[44] Kuypers F.A. (2008). Red Cell Membrane Lipids in Hemoglobinopathies. Curr. Mol. Med..

[45] Striesow J., Lackmann J.-W., Ni Z., Wenske S., Weltmann K.-D., Fedorova M., von Woedtke T., Wende K. (2019). Oxidative modification of skin lipids by cold atmospheric plasma (CAP): A standardizable approach using RP-LC/MS2 and DI-ESI/MS2. Chem. Phys. Lipids.

[46] Bassereau P., Pincet F. (1997). Quantitative Analysis of Holes in Supported Bilayers Providing the Adsorption Energy of Surfactants on Solid Substrate. Langmuir.

[47] Benz M., Gutsmann T., Chen N., Tadmor R., Israelachvili J. (2004). Correlation of AFM and SFA Measurements Concerning the Stability of Supported Lipid Bilayers. Biophys. J..

[48] Marsh D. (2010). Liquid-ordered phases induced by cholesterol: A compendium of binary phase diagrams. Biochim. Biophys. Acta (BBA) Biomembr..

[49] Ravandeh M., Coliva G., Kahlert H., Azinfar A., Helm C.A., Fedorova M., Wende K. (2021). Protective Role of Sphingomyelin in Eye Lens Cell Membrane Model against Oxidative Stress. Biomolecules.

[50] Ravandeh M., Kahlert H., Jablonowski H., Lackmann J.-W., Striesow J., Hernández V.A., Wende K. (2020). A combination of electrochemistry and mass spectrometry to monitor the interaction of reactive species with supported lipid bilayers. Sci. Rep..

[51] Graves D.B. (2014). Reactive Species from Cold Atmospheric Plasma: Implications for Cancer Therapy. Plasma Process. Polym..

[52] de Meyer F., Smit B. (2009). Effect of cholesterol on the structure of a phospholipid bilayer. Proc. Natl. Acad. Sci. USA.

[53] Van Der Paal J., Verheyen C., Neyts E.C., Bogaerts A. (2017). Hampering Effect of Cholesterol on the Permeation of Reactive Oxygen Species through Phospholipids Bilayer: Possible Explanation for Plasma Cancer Selectivity. Sci. Rep..

[54] Bogaerts A., Yusupov M., Razzokov J., Van der Paal J. (2019). Plasma for cancer treatment: How can RONS penetrate through the cell membrane? Answers from computer modeling. Front. Chem. Sci. Eng..

[55] Valentine M.L., Waterland M.K., Fathizadeh A., Elber R., Baiz C.R. (2021). Interfacial Dynamics in Lipid Membranes: The Effects of Headgroup Structures. J. Phys. Chem. B.

[56] Slotte J.P. (2016). The importance of hydrogen bonding in sphingomyelin’s membrane interactions with co-lipids. Biochim. Biophys. Acta (BBA)-Biomembr..

[57] Slotte J.P. (2013). Biological functions of sphingomyelins. Prog. Lipid Res..

[58] Madrid E., Horswell S.L. (2013). Effect of Headgroup on the Physicochemical Properties of Phospholipid Bilayers in Electric Fields: Size Matters. Langmuir.

[59] Pasenkiewicz-Gierula M., Takaoka Y., Miyagawa H., Kitamura A.K., Kusumi A. (1997). Hydrogen Bonding of Water to Phosphatidylcholine in the Membrane As Studied by a Molecular Dynamics Simulation: Location, Geometry, and Lipid−Lipid Bridging via Hydrogen-Bonded Water. J. Phys. Chem. A.

[60] Ripa I., Andreu S., López-Guerrero J.A., Bello-Morales R. (2021). Membrane Rafts: Portals for Viral Entry. Front. Microbiol..

[61] Verlackt C.C.W., Neyts E., Bogaerts A. (2017). Atomic scale behavior of oxygen-based radicals in water. J. Phys. D. Appl. Phys..

[62] Wong-Ekkabut J., Xu Z., Triampo W., Tang I.-M., Tieleman D.P., Monticelli L. (2007). Effect of Lipid Peroxidation on the Properties of Lipid Bilayers: A Molecular Dynamics Study. Biophys. J..

[63] Riske K.A., Sudbrack T.P., Archilha N.L., Uchoa A.F., Schroder A.P., Marques C.M., Baptista M.S., Itri R. (2009). Giant Vesicles under Oxidative Stress Induced by a Membrane-Anchored Photosensitizer. Biophys. J..

[64] Gellert F., Ahrens H., Helm C.A. (2020). Oxidation of Unsaturated Phospholipids: A Monolayer Study. Langmuir.

[65] Bahja J., Dymond M.K. (2021). Does membrane curvature elastic energy play a role in mediating oxidative stress in lipid membranes?. Free. Radic. Biol. Med..

[66] Tero R., Yamashita R., Hashizume H., Suda Y., Takikawa H., Hori M., Ito M. (2016). Nanopore formation process in artificial cell membrane induced by plasma-generated reactive oxygen species. Arch. Biochem. Biophys..

[67] Tsubone T.M., Junqueira H.C., Baptista M.S., Itri R. (2018). Contrasting roles of oxidized lipids in modulating membrane microdomains. Biochim. Biophys. Acta (BBA) Biomembr..

[68] O’Donnell V.B. (2011). Mass spectrometry analysis of oxidized phosphatidylcholine and phosphatidylethanolamine. Biochim. Biophys. Acta (BBA) Mol. Cell Biol. Lipids.

[69] Madrid E., Horswell S.L. (2018). The electrochemical phase behaviour of chemically asymmetric lipid bilayers supported at Au (111) electrodes. J. Electroanal. Chem..

[70] Mann M.S., Tiede R., Gavenis K., Daeschlein G., Bussiahn R., Weltmann K.-D., Emmert S., Von Woedtke T., Ahmed R. (2016). Introduction to DIN-specification 91315 based on the characterization of the plasma jet kINPen® MED. Clin. Plasma Med..

[71] Reuter S., von Woedtke T., Weltmann K.D. (2018). The kINPen-a review on physics and chemistry of the atmospheric pressure plasma jet and its applications. J. Phys. D Appl. Phys..

[72] Linxiang L., Abe Y., Nagasawa Y., Kudo R., Usui N., Imai K., Mashino T., Mochizuki M., Miyata N. (2004). An HPLC assay of hydroxyl radicals by the hydroxylation reaction of terephthalic acid. Biomed. Chromatogr..

[73] Bekeschus S., Wende K., Hefny M.M., Rödder K., Jablonowski H., Schmidt A., von Woedtke T., Weltmann K.-D., Benedikt J. (2017). Oxygen atoms are critical in rendering THP-1 leukaemia cells susceptible to cold physical plasma-induced apoptosis. Sci. Rep..

[74] Bekeschus S., Schmidt A., Niessner F., Gerling T., Weltmann K.-D., Wende K. (2017). Basic Research in Plasma Medicine—A Throughput Approach from Liquids to Cells. J. Vis. Exp..

[75] Nasri Z., Bruno G., Bekeschus S., Weltmann K.-D., von Woedtke T., Wende K. (2020). Development of an electrochemical sensor for in-situ monitoring of reactive species produced by cold physical plasma. Sens. Actuators B Chem..

[76] Liebisch G., Fahy E., Aoki J., Dennis E.A., Durand T., Ejsing C., Fedorova M., Feussner I., Griffiths W.J., Koefeler H. (2020). Update on LIPID MAPS Classification, Nomenclature and Shorthand Notation for MS-derived Lipid Structures. J. Lipid Res..

[77] Nasri Z., Memari S., Wenske S., Clemen R., Martens U., Delcea M., Bekeschus S., Weltmann K., von Woedtke T., Wende K. (2021). Singlet-Oxygen-Induced Phospholipase A 2 Inhibition: A Major Role for Interfacial Tryptophan Dioxidation. Chem. A Eur. J..

[78] Clemen R., Freund E., Mrochen D., Miebach L., Schmidt A., Rauch B.H., Lackmann J., Martens U., Wende K., Lalk M. (2021). Gas Plasma Technology Augments Ovalbumin Immunogenicity and OT-II T Cell Activation Conferring Tumor Protection in Mice. Adv. Sci..

[79] Jackman J.A., Knoll W., Cho N.-J. (2012). Biotechnology Applications of Tethered Lipid Bilayer Membranes. Materials.

[80] Denisov I.G., Sligar S.G. (2017). Nanodiscs in Membrane Biochemistry and Biophysics. Chem. Rev..

