# Supplementary material for: Insight into the Impact of Oxidative Stress on the Barrier Properties of Lipid Bilayer Models
Source: Int J Mol Sci. 2022 May 25;23(11):5932. doi: 10.3390/ijms23115932 (PMC9180489; doi:10.3390/ijms23115932)
Supplement: Supplementary file 1 [file ijms-23-05932-s001.zip › ijms-1735473-supplementary.pdf]

# The impact of oxidative stress on the barrier properties of lipid bilayer models

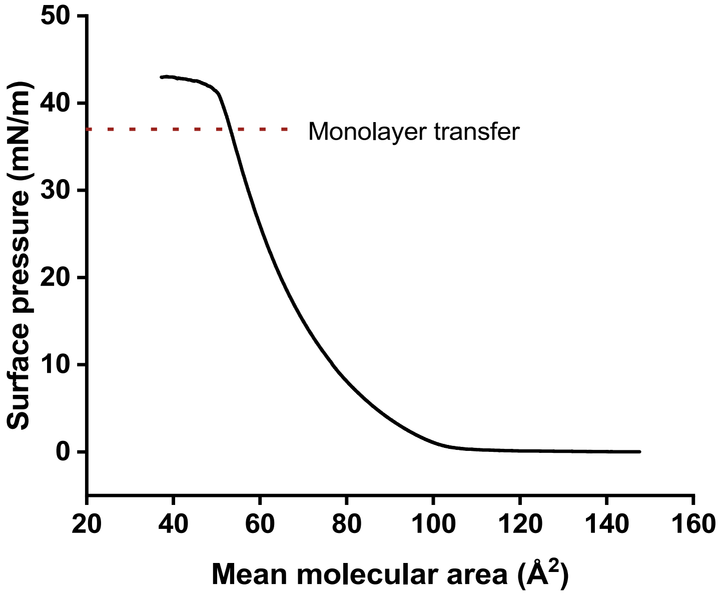


Figure S1. $\pi$-A isotherm of DOPE + DOPS + 10% cholesterol obtained on a pure water surface in a nitrogen atmosphere at 22 °C.

Table S1. Target Pressures for the transfer of monolayers with different lipid compositions with Langmuir-Blodgett and Langmuir-Schaefer techniques

| Lipid composition | Target pressure (mN/m) |
| --- | --- |
| DOPE + DOPS + 10% cholesterol | 37 |
| POPC + SM(d18:1/18:0) + 10% cholesterol | 37 |
| DOPE + DOPS | 38 |
| POPC + SM(d18:1/18:0) | 36 |
| POPC | 35 |
| SM(d18:1/18:0) | 41 |
| DOPE | 37 |
| DOPS | 38 |

Table S2. Retention time (RT) analysis of POPC

| lipid peroxidation products | Formula neutral | Obs. Mass $[M+CH_3COO]^-$ | RT [min] |
|---|---|---|---|
| POPC 16:0/18:1;OH,OOH or 16:0/18:0;3O | $C_{42}H_{82}NO_{11}P$ | 866.5768 | 6.77 |
| POPC 16:0/18:0;oxo or Ep | $C_{42}H_{82}NO_9P$ | 834.5864 | 6.90/7.02 |
| POPC 16:0/8:0;oxo | $C_{32}H_{62}NO_9P$ | 694.4304 | 7.33 |
| POPC 16:0/9:0;oxo | $C_{33}H_{64}NO_9P$ | 708.4464 | 7.26 |
| POPC 16:0/9:0;COOH | $C_{33}H_{64}NO_{10}P$ | 664.4199 [-H] | 8.36/8.50 |

Table S3. Retention time (RT) analysis of DOPE

| lipid peroxidation products | Formula neutral | Obs. Mass $[M-H]^-$ | RT [min] |
|---|---|---|---|
| DOPE 18:1/9:0;oxo | $C_{32}H_{60}NO_9P$ | 632.3936 | 6.70 |
| DOPE 18:1/9:0;COOH | $C_{32}H_{60}NO_{10}P$ | 634.3727 | 7.96 |
| DOPE 18:1/18:1;oxo | $C_{41}H_{76}NO_9P$ | 756.5177 | 6.41 |
| DOPE 18:1_0:0 | $C_{23}H_{46}NO_7P$ | 478.2939 | 7.57/7.72 |
| DOPE 18:1/18:0;oxo or Ep | $C_{41}H_{78}NO_9P$ | 758.5346 | 6.41/6.53 |
| DOPE 18:1/18:1;oxo(2) | $C_{41}H_{74}NO_{10}P$ | 770.4976 | 6.51 |

Table S4. Retention time (RT) analysis of DOPS

| lipid peroxidation products | Formula neutral | Obs. Mass $[M-H]^-$ | RT [min] |
|---|---|---|---|
| DOPS 18:1/18:1;GPAA | $C_{41}H_{75}O_{10}P$ | 757.4835 | 6.39 |
| DOPS 18:1/9:0;oxo | $C_{33}H_{60}NO_{11}P$ | 676.3836 | 7.12 |
| DOPS 18:1/18:1;$(OOH)_2$ or $(OH)_4$ or DOPS 18:1/18:0;$(OH)_2$;$(oxo)_2$ | $C_{42}H_{78}NO_{14}P$ | 850.5082 | 6.94 |
| DOPS 18:1/18:1;$O_3$ | $C_{42}H_{78}NO_{13}P$ | 834.5135 | 6.98 |
| DOPS 18:1/18:1;OOH, oxo | $C_{42}H_{76}NO_{13}P$ | 832.4982 | 6.92 |
| DOPS 18:1/18:1;OH, oxo | $C_{42}H_{76}NO_{12}P$ | 816.5048 | 7.00 |
| DOPS 18:1/18:0;oxo or Ep | $C_{42}H_{78}NO_{11}P$ | 802.5229 | 6.84 |
| DOPS 18:1/18:1;oxo | $C_{42}H_{76}NO_{11}P$ | 800.5088 | 6.71 |

Table S5. Retention time (RT) analysis of SM (d18:1/18:0)

| lipid peroxidation products | Formula neutral | Obs. Mass $[M+CH_3COO]^-$ | RT [min] |
|---|---|---|---|
| SM 18:0/18:1;OH | $C_{41}H_{83}N_2O_7P$ | 805.6076 | 7.74 |
| SM 18:0/18:1;oxo | $C_{41}H_{81}N_2O_7P$ | 803.592 | 7.57 |

- POPC 16:0/9:0;oxo

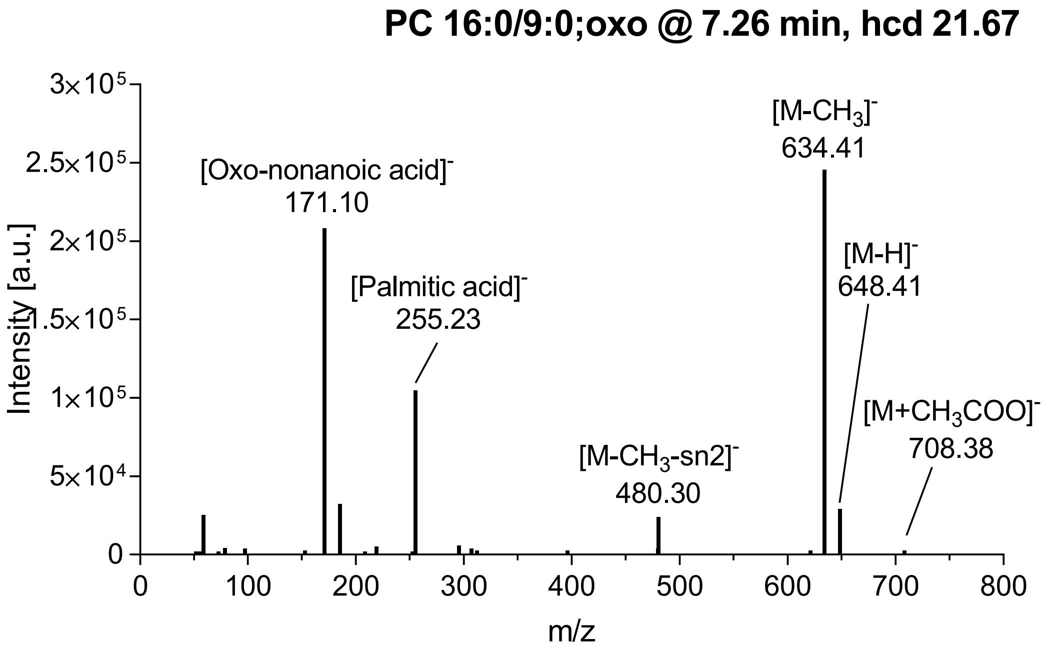


- POPC 16:0/9:0;COOH

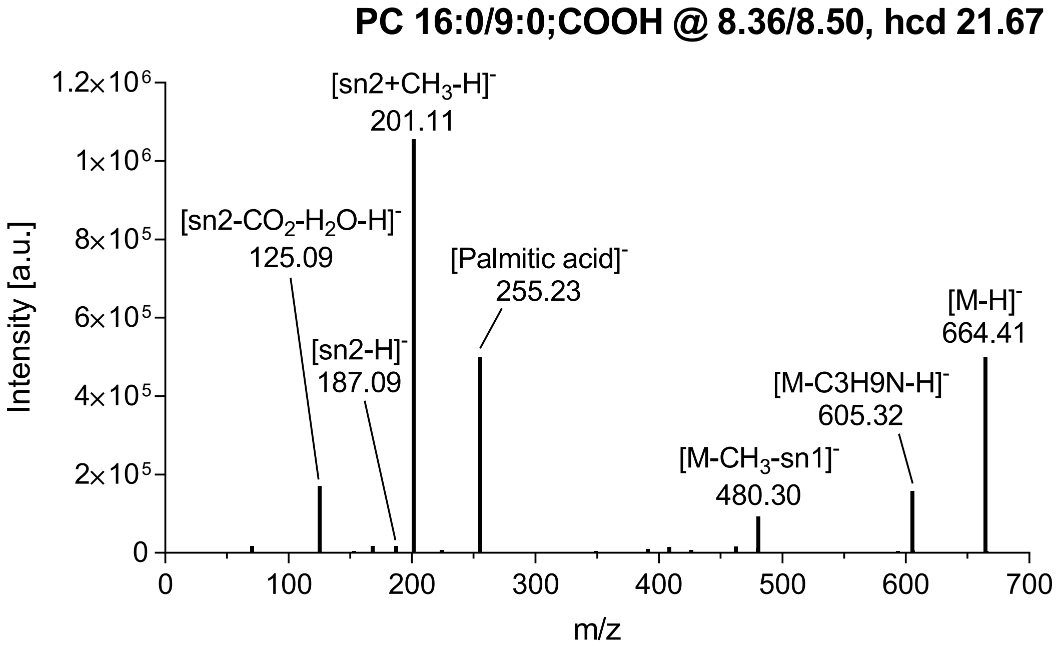

- DOPE 18:1/9:0;oxo

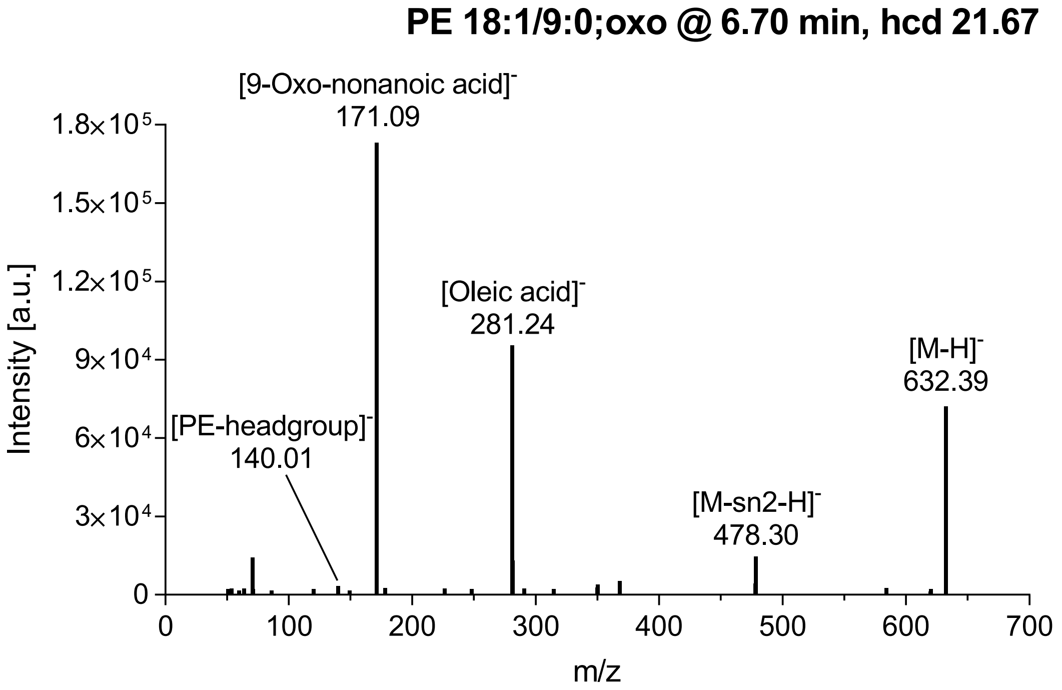


- DOPE 18:1/18:1;oxo

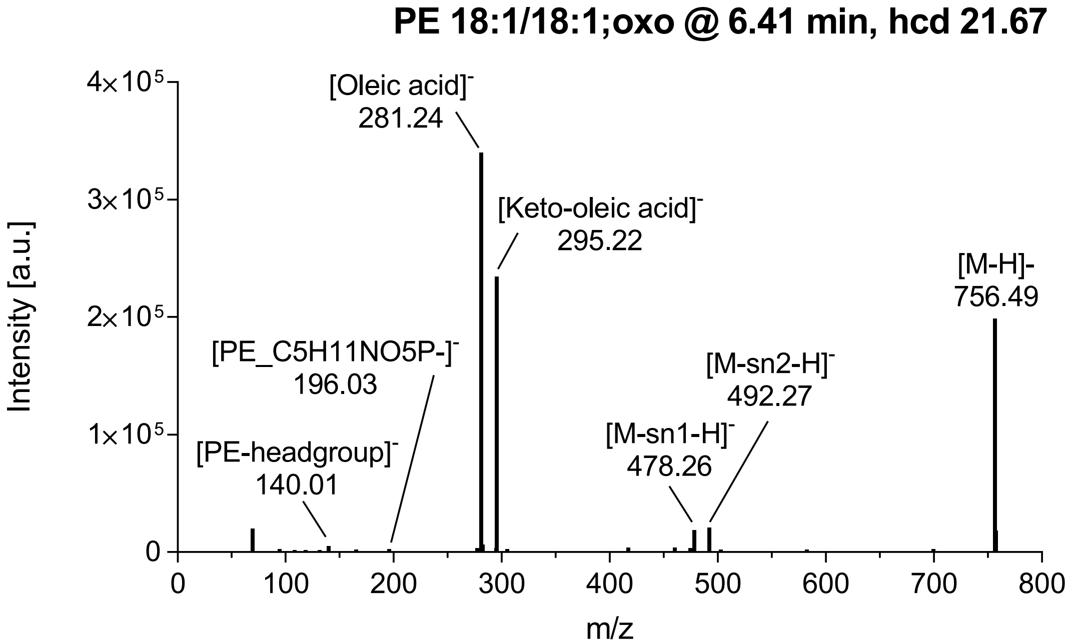

- DOPS 18:1/18:1;GPAA

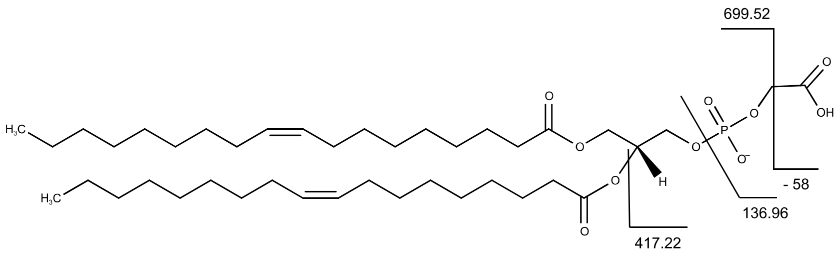


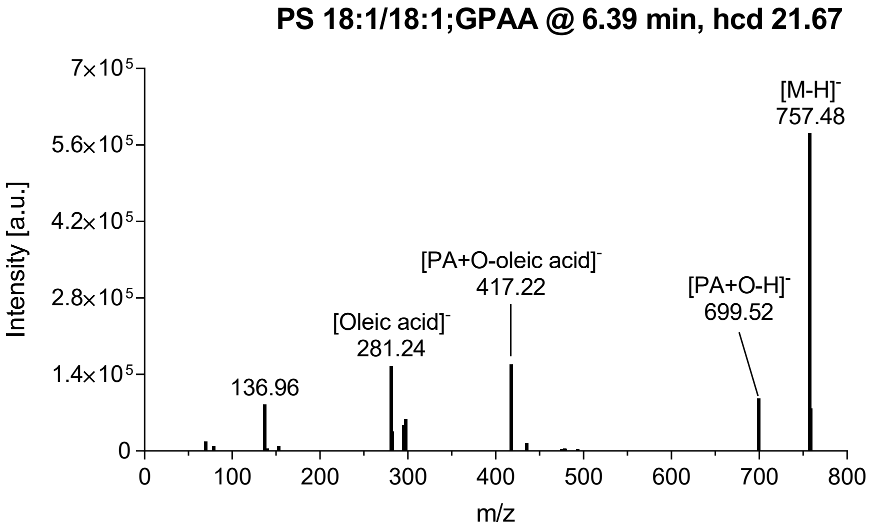


- DOPS 18:1/18:0;oxo or Ep

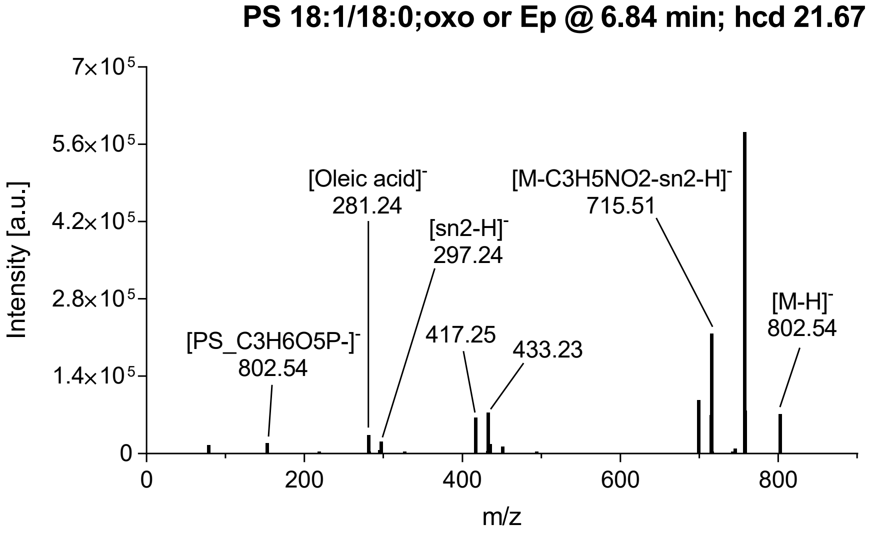


Figure S2. MS[2] fragmentation pattern of selected LPPs.
